# Sharing the wealth: The versatility of proteins targeted to peroxisomes and other organelles

**DOI:** 10.3389/fcell.2022.934331

**Published:** 2022-09-26

**Authors:** Elena Bittner, Thorsten Stehlik, Johannes Freitag

**Affiliations:** Department of Biology, Philipps-University Marburg, Marburg, Germany

**Keywords:** dual targeting, Endoplasmic reticulum, peroxisome, targeting signal, Mitochondria

## Abstract

Peroxisomes are eukaryotic organelles with critical functions in cellular energy and lipid metabolism. Depending on the organism, cell type, and developmental stage, they are involved in numerous other metabolic and regulatory pathways. Many peroxisomal functions require factors also relevant to other cellular compartments. Here, we review proteins shared by peroxisomes and at least one different site within the cell. We discuss the mechanisms to achieve dual targeting, their regulation, and functional consequences. Characterization of dual targeting is fundamental to understand how peroxisomes are integrated into the metabolic and regulatory circuits of eukaryotic cells.

## Origin of peroxisomes

Peroxisomes are eukaryotic organelles containing enzymes for the breakdown of reactive oxygen species and fatty acids (Poirier et al., 2006; Wanders, 2014). Peroxisomes may have no direct endosymbiotic origin, are unlike mitochondria and plastids devoid of nucleic acids but show a certain degree of autonomy, and contain dedicated systems for protein import (Gabaldón, 2010; Smith and Aitchison, 2013). One scenario is their emergence from the endoplasmic reticulum (ER) to reduce the detrimental effects of reactive oxygen species on ER protein homeostasis (Gabaldón, 2014). This idea is supported by findings indicating that the import machinery for peroxisomal matrix proteins is similar to the ERAD system known to control the export of misfolded proteins from the ER (Gabaldón et al., 2006; Schlüter et al., 2006).

According to a different opinion, peroxisomes are derivatives of mitochondria, since both organelles are sites of fatty acid breakdown. Peroxisomes may have emerged during the evolution of ancient eukaryotic cells to reduce the ROS burden of their progenitor (Speijer, 2017). Phylogenetic analysis revealed a bacterial origin of three out of four β-oxidation enzymes (Bolte et al., 2015). This led the authors to propose that at least the present form of peroxisomes emerged after mitochondria since a major catabolic pathway of peroxisomes likely originated from this organelle (Bolte et al., 2015). Mitochondrial enzymes probably became retargeted to peroxisomes over time, and some of these still remained dually localized (Gabaldón, 2018). Indeed, recent work shows that the early diverged cryptophyte *Guillardia theta* contains peroxisomes, but enzymes for fatty acid oxidation seem to be only mitochondrial (Vasilev et al., 2022). Independent of the actual evolutionary scenario, both the ER and mitochondria likely contributed to the proteome of modern peroxisomes and still sustain their biogenesis.

## Peroxisome functions

The biochemical functions of peroxisomes are versatile. We only provide an overview and mainly refer to review articles focused on a more detailed explanation of peroxisomes from different species. The coupled degradation of fatty acids and H_2_O_2_ is a prominent task, and peroxisomes owe their name to this process (De Duve and Baudhuin, 1966; Poirier et al., 2006). Remarkably, in several parasitic species including trypanosomes peroxisomes house the enzymes for glycolysis (Haanstra et al., 2016). This already highlights a fascinating feature of the organelle—it is highly adaptable to a specific lifestyle. Another example is the metabolism of methylotrophic yeasts that break down methanol and harbor this oxidative process inside of the peroxisome (van der Klei and Veenhuis, 2006b). Yeasts and filamentous fungi contain a large variety of biosynthetic pathways inside of peroxisomes including enzymes involved in the production of antibiotics, biotin, surface-active glycolipids, and siderophores (Meijer et al., 2010; Magliano et al., 2011; Tanabe et al., 2011; Gründlinger et al., 2013; Freitag et al., 2014; Stehlik et al., 2014). Furthermore, peroxisomes are important for virulence of several human- and plant-pathogenic fungi (Kretschmer et al., 2012a; Falter and Reumann, 2022).

Peroxisomes are essential for the regular development of humans, and mutations in peroxisomal proteins are associated with severe diseases including the Zellweger syndrome (Wanders, 2014). Besides their prominent function in fatty acid catabolism, mammalian peroxisomes are required for further processes such as the production of ether lipids and bile acids or metabolism of amino acids including D-amino acids (Wanders and Waterham, 2006; Wanders et al., 2016). Furthermore, peroxisomes play an important role in the development of the brain and their dysfunction may contribute to neurological pathologies including amyotrophic lateral sclerosis and Alzheimer’s disease (Berger et al., 2016). More recent evidence supports a major function of peroxisomes in regulating the response of the immune system to pathogenic attack in several animals (Odendall et al., 2014; Di Cara et al., 2017; Di Cara, 2020). Plant peroxisomes are critical for oil mobilization during early seedling development and play a role in the metabolism of the toxic by-product 2-phosphoglycolate derived from an O_2_-consuming side reaction of RuBisCO. 2-Phosphoglycolate needs to be removed from the plastid, where it can inhibit several enzymes and is recycled into 3-phosphoglycerate in a series of reactions involving enzymatic reactions in peroxisomes and mitochondria (Reumann and Weber, 2006; Hu et al., 2012; Dellero et al., 2016; Pan et al., 2020). Besides their metabolic tasks, peroxisomes are also emerging as cellular signaling platforms (Tripathi and Walker, 2016). In addition, they can act as proviral and antiviral organelles depending on the type of virus (Ferreira et al., 2022). Given this remarkable variability, it is likely that many more functions of peroxisomes remain elusive, which may often be specific to a particular organism.

## Protein targeting to peroxisomes

To attach to a defined organelle, proteins usually possess targeting signals, which act as a molecular zip code (Blobel and Sabatini, 1971). Proteins designated for the peroxisomal matrix mostly contain C-terminal or N-terminal sequence motifs termed peroxisomal targeting signal type I (PTS1) or type II (PTS2) (Kunze et al., 2015; Francisco et al., 2017; Walter and Erdmann, 2019; Kunze, 2020; Bürgi et al., 2021). These signals are recognized and bound by soluble targeting factors in the cytosol. Cargo proteins can be imported in a folded state and even as oligomers (Francisco et al., 2017; Walter and Erdmann, 2019). Very large particles have been shown to be imported into the lumen of the organelle revealing a flexible import pore (Walton et al., 1995; Yang et al., 2018). Proteins without a defined signal can enter the peroxisome in complex with canonical cargo (Islinger et al., 2009; Schueren et al., 2014; Effelsberg et al., 2015; Al-Saryi et al., 2017a; Gabay-Maskit et al., 2020). This piggyback import may be a hallmark of import pathways, which accept folded or partially folded clients such as the nucleus and the peroxisome. Several proteins are known to contain elements that enable binding to the targeting factor Pex5 in the cytosol although they lack a classical PTS1 (van der Klei and Veenhuis, 2006a; Kempiński et al., 2020; Rosenthal et al., 2020; Yifrach et al., 2021). Peroxisomal membrane proteins (PMPs) can be directly inserted into the peroxisomal membrane aided by the chaperone Pex19 and the transmembrane protein Pex3, but some can also be sorted *via* the ER (for a review, see Kim and Hettema, 2015).

## Mechanisms to achieve the dual localization of proteins

We have previously discussed mechanisms involved in dual targeting of peroxisomal proteins in greater detail (Ast et al., 2013). Therefore, they are only briefly discussed, and instead, the focus of this work is on more recent findings on the plethora of dually targeted proteins and their potential function. We provide single chapters focusing on proteins targeted to peroxisomes and at least one other organelle with a major focus on yeasts and filamentous fungi. In addition, we showcase parallels to other eukaryotes.

Dual targeting can be achieved through very different mechanisms. Gene duplication and subsequent development of isoforms with import signals for only one cellular compartment are often found in *Saccharomyces cerevisiae*, presumably due to the genome duplications, which happened during its evolution (Kellis et al., 2004; Yogev et al., 2011; Ast et al., 2013). Polypeptides with different targeting signals can also be generated from a single gene (Yogev et al., 2011; Ast et al., 2013), e.g., from alternative transcripts, alternative splicing (Natsoulis et al., 1986; Clausmeyer et al., 1999; Freitag et al., 2012; Strijbis et al., 2012), programmed readthrough of stop codons (for a review, see Schueren and Thoms, 2016), and noncanonical translation initiation (Monteuuis et al., 2019; Kremp et al., 2020). In addition, proteins can contain ambiguous targeting signals at their N-termini, which enable sorting into two cellular compartments. This is particularly prominent in plants for those proteins required in mitochondria and plastids—two organelles, which use a related protein import pathway involving N-terminal targeting signals and translocation of unfolded proteins (Carrie et al., 2009). We also discuss the dual targeting of proteins that contain N-terminal targeting signals, e.g., for mitochondria or the ER in combination with a C-terminal PTS1. It was suggested previously that the localization of these proteins might be dictated by the N-terminal signal since it can be bound by respective targeting factors during translation before the C-terminal PTS1 becomes accessible (Ast et al., 2013; Kunze and Berger, 2015). However, a recent study uncovered that proteins with competing N-terminal and C-terminal targeting signals localize in peroxisomes and mitochondria (Stehlik et al., 2020). Low efficiency or unusual peroxisomal targeting signals often provoke partial cytosolic retention due to low import rates or other reasons, e.g., the modification of the peroxisomal targeting machinery (Ast et al., 2013; Okumoto et al., 2020). Another focus of our review is on peroxisomal membrane proteins (PMPs) and membrane-associated proteins, which follow variable transit routes.

## Typical enzymes with a role in multiple cellular compartments

**FIGURE 1:**
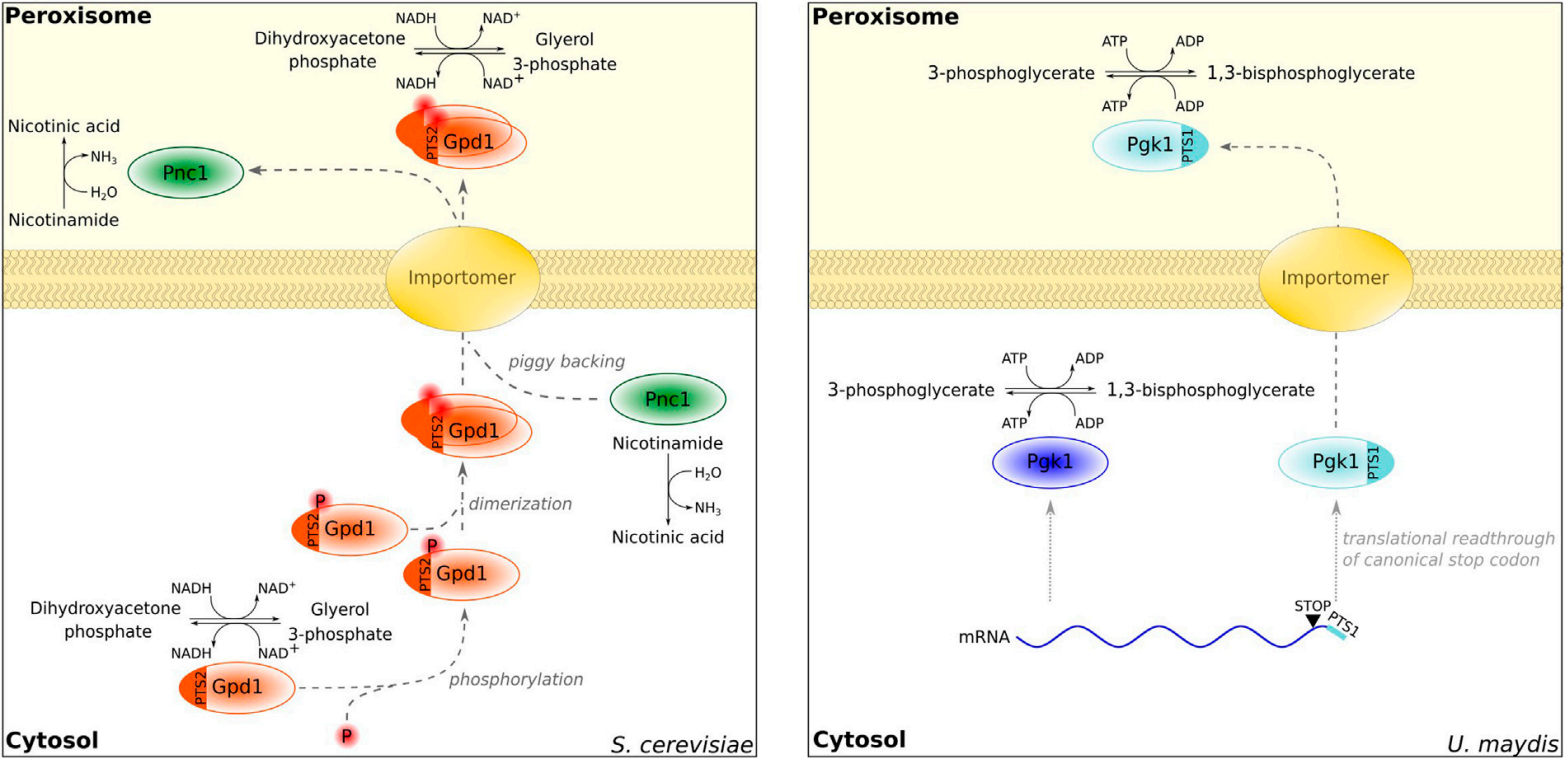
Examples of proteins localized in the cytosol and peroxisomes. Gpd1 distribution is controlled by phosphorylation [left; Jung et al. (2010)]. In addition, Gpd1 regulates the sorting of Pnc1 *via* piggybacking (Effelsberg et al. (2015), Kumar et al. (2016), Al-Saryi et al. (2017b)). An extended isoform of Pgk1 derived from translational readthrough is localized in peroxisomes in *Ustilago maydis* [right; Freitag et al. (2012)].

One critical type of enzymes required inside of peroxisomes and in other compartments are NADH-dependent dehydrogenases. They can assist peroxisomal NAD^+^ regeneration, e.g., during β-oxidation. The reduced substrate can be translocated into the cytosol and exchanged with an oxidized molecule giving rise to a redox shuttle system (Visser et al., 2007). The existence of a peroxisomal redox shuttle was first demonstrated in *S. cerevisiae*—a peroxisomal isoenzyme of malate dehydrogenase (Mdh3p) containing a PTS1 was shown to be involved (Van Roermund et al., 1995). Malate can be generated from oxaloacetate enabling the reoxidation of NADH. The small molecules are thought to pass the peroxisomal membrane, albeit it is not fully understood if transporters or size-selective pore-forming proteins are involved (for a review, see Chornyi et al., 2021). Recently, it was shown that a second malate dehydrogenase Mdh2p from *S. cerevisiae* involved in glyoxylate metabolism binds Mdh3p and can enter peroxisomes *via* piggyback import resulting in dual localization (Gabay-Maskit et al., 2020). It is yet unclear how cells exactly benefit from having two MDH enzymes inside of peroxisomes, but a possible answer is their different activities (Steffan and McAlister-Henn, 1992; Gabay-Maskit et al., 2020). In addition to the described malate–oxaloacetate shuttle, *S. cerevisiae* contains a second shuttle system relying on the glycerol-3-phosphate dehydrogenase Gpd1p, which catalyzes the interconversion of glycerol-3-phosphate and dihydroxyacetone phosphate. Gpd1p is dually localized in the cytosol and in peroxisomes (Jung et al., 2010) (Figure 1). Mdh3p and Gpd1p exhibit redundant functions for NAD shuttling in conditions that require biosynthesis of lysine, the last step of which occurs inside of yeast peroxisomes (Al-Saryi et al., 2017a). Why cells employ multiple shuttle systems for NAD^+^/NADH and how this functionally connects peroxisomal metabolism to the metabolism of the entire cell requires further investigation. The presence of multiple systems, however, seems to be common. Many fungi contain a peroxisomal isoform of the NAD^+^-dependent glycolytic enzyme glyceraldehyde-3-phosphate dehydrogenase (GAPDH) derived from alternative splicing or stop codon readthrough. Genetic data point to overlapping functions of peroxisomal GAPDH, GPD, and MDH presumably for the regulation of NAD^+^/NADH balance (Freitag et al., 2012).

In mammalian cells, programmed stop codon readthrough triggers the synthesis of extended PTS1-containing isoforms of lactate dehydrogenase LDHB and malate dehydrogenase MDH1 (Schueren et al., 2014; Stiebler et al., 2014). This is in accordance with at least two pathways to control the NAD^+^/NADH ratio. Stop codon readthrough is a widespread mechanism to regulate the dual localization of central metabolic enzymes in fungi and animals; e.g., a peroxisomal isoform of the glycolytic enzyme phosphoglycerate kinase contains a readthrough-derived PTS1 (Freitag et al., 2012; Figure 1). Other prominent enzymes occurring in many cellular compartments are inorganic pyrophosphatases—several enzymes harbor putative PTS1-containing extensions, which can be activated by stop codon readthrough. These include the *Caenorhabditis elegans* Pyp-1 protein and PAP1 or PAP2 from different mammals (Stiebler et al., 2014). Inorganic pyrophosphatase is a central enzyme rendering energy-dependent reactions throughout the cell virtually irreversible (Kornberg, 1962) and may also speed up peroxisomal metabolism.

Why is stop codon readthrough such a prominent mechanism for the generation of peroxisomal isoforms? First, a trivial reason is the C-terminal position of PTS1—a simple prerequisite for this mechanism to activate a hidden PTS1. Furthermore, readthrough rates are low but seem to be sufficient to enable enzyme supply for the relatively small peroxisomal compartment in concentrations high enough to satisfy demands. Finally, the region behind the stop codon could be regarded as a playground for evolution and partial peroxisomal localization can be easily tested and rejected again. This may explain the previously observed patchy distribution of readthrough sites coupled to PTS1 motifs among different eukaryotic species (Freitag et al., 2012; Dunn et al., 2013; Stiebler et al., 2014; Hofhuis et al., 2016).

Peroxisomal NADPH turnover is involved in the oxidation of fatty acids with a cis double bond at an even position (Poirier et al., 2006). Early work in *S. cerevisiae* demonstrated a role of an isocitrate dehydrogenase as part of a peroxisomal NADPH shuttle (van Roermund et al., 1998). Isocitrate is oxidized to alpha-ketoglutarate and CO_2_ leading to the formation of NADPH. Idp1p is the mitochondrial isoform, Idp2p is the cytosolic isoform, and Idp3p is the peroxisomal isoform (Haselbeck and McAlister-Henn, 1991; Haselbeck and McAlister-Henn, 1993; van Roermund et al., 1998). In contrast, peroxisomal, mitochondrial, and cytosolic isocitrate dehydrogenases are derived from a single gene in the filamentous fungus *Neurospora crassa* (Szewczyk et al., 2001). In *Arabidopsis thaliana*, several enzymes of the pentose phosphate pathway including an isoform of the NADPH-dependent enzyme 6-phosphogluconate dehydrogenase (PGD) reside in peroxisomes and are critical for development (Corpas et al., 1998; Meyer et al., 2011; Hölscher et al., 2016). Dually targeted peroxisomal isoforms of PGD can be generated by alternative splicing or noncanonical translation initiation in different fungi (Strijbis et al., 2012; Kremp et al., 2020). Recently, in *A. thaliana* a transport protein for glucose-6-phosphate has been identified that reaches peroxisomes *via* the ER but is also found in plastids (Baune et al., 2020).

Additional carbohydrate-metabolizing enzymes contain cryptic or low-efficiency peroxisomal targeting signals in several fungi including not only glycolytic/gluconeogenetic enzymes but also enzymes of the non-oxidative part of the pentose phosphate pathway (Freitag et al., 2012; Freitag et al., 2018). This points to many molecular links of the peroxisomal metabolism to the central carbohydrate metabolism. In several fungal species, optimal growth on glucose medium requires intact peroxisomes—even on full medium in the logarithmic growth phase (Idnurm et al., 2007; Freitag et al., 2012; Camões et al., 2015; Ast et al., 2022). Whether these growth phenotypes are directly related to the dual targeting of glycolytic enzymes or pentose phosphate pathway enzymes is yet elusive.

## Dually targeted proteins—the dark matter of the peroxisomal proteome

Minor destinations of proteins are very likely to be missed. A recent systematic study in *S. cerevisiae* uncovered many novel proteins residing in peroxisomes, as well as in other organelles or in the cytosol (Yifrach et al., 2021). Using systematic metabolomics analysis of mutants and overexpression strains, the authors suggest that several of these have potential peroxisomal functions. Among the newly identified dually localized proteins are direct substrates of Pex5, which lack a classical PTS1. This reveals a greater substrate repertoire of this targeting factor (Yifrach et al., 2021). Other work also points to the occurrence of many unusual variations of the PTS1 motif, which are recognized by Pex5 in several fungi (Freitag et al., 2012; Camões et al., 2015; Nötzel et al., 2016). An unexpected example is sequence motifs resembling a PTS1, which are located near the C-terminus of a protein rather than at its end. This type of signal was suggested to be responsible for peroxisomal localization of catalase from pumpkin and for human ataxia telangiectasia–mutated (ATM) kinase (Kamigaki et al., 2003; Zhang J. et al., 2015). It is still unclear how PTS1 motifs, which do not reside at the C-terminus, are recognized by Pex5 as this is thought to be a structural prerequisite for binding (Fodor et al., 2015).

An interesting example identified by Yifrach et al. (2021) is dually localized subunits of the GID (glucose-induced degradation-deficient) complex, which regulate the stability of fructose-1,6-bisphosphatase (FBP). The targeting of GID complex proteins to peroxisomes was found to increase FBP levels probably enhancing gluconeogenesis (Yifrach et al., 2021). These data indicate a novel function of peroxisomes in regulating carbohydrate mechanism upon glucose limitation. Similarly, in different yeasts nicotinamidase Pnc1 is targeted to peroxisomes more efficiently in response to certain stresses—this is achieved through hitchhiking on the PTS2 protein Gpd1 (Anderson et al., 2003; Effelsberg et al., 2015; Kumar et al., 2016; Al-Saryi et al., 2017b) (Figure 1). Sequestration of a protein inside of the peroxisomal lumen to remove it from its site of action anywhere in the cell may be a more widespread function of peroxisomes. They seem to be promiscuous sites among the cellular organelles and tolerate many different proteins and activities. Even the efficient production of toxic compounds was successfully engineered aided by the peroxisomal targeting of respective enzymes (Grewal et al., 2021). For each of the dually localized proteins, it will be critical to assess whether they fulfill a specific function inside of peroxisomes or whether their localization reflects a strategy to sequester them from a different cellular compartment.

Many functions of peroxisomes relying on dually targeted proteins are likely to remain established. Surprisingly, in *Aspergillus nidulans* the microtubule-organizing protein ApsB is partially localized to a subpopulation of peroxisomes, but its role at this location has not been understood (Zekert et al., 2010). Several proteins from *A. thaliana* show a dual localization to peroxisomes and to other compartments, often due to PTS1 motifs with low import efficiency (Reumann et al., 2007; Kataya and Reumann, 2010; Lingner et al., 2011). Interestingly, protein kinases were identified that often contain unusual but functional PTS1 motifs (Kataya et al., 2022). These findings are of particular interest as little is known about the phosphorylation-dependent regulation of enzymes inside of the peroxisomal lumen. More data are needed to assess which kinases play a crucial role in regulating the activity of the peroxisomal proteome. Particularly, the fact that some kinases contain transmembrane domains is puzzling with regard to the classical view of peroxisomal matrix protein import (Walter and Erdmann, 2019). Recent data, however, point to a function of the peroxisomal matrix protein import machinery in membrane protein translocation (Martenson et al., 2020). Together, all the mentioned studies emphasize a plethora of diverse peroxisomal proteins, for which a functional characterization is lacking so far. We expect more surprises concerning the proteome composition of peroxisomes in the future.

## Peroxisomes—organelles in a twilight zone

Peroxisomes have been adapted for multiple purposes during evolution, e.g., as a seal for septal pores in fungal hyphae, as sites for methanol breakdown in different yeast, or as glycolytic organelles in trypanosomes (Jedd and Chua, 2000; van der Klei and Veenhuis, 2006b; Liu et al., 2011; Haanstra et al., 2016). Why do peroxisomes show this high degree of functional flexibility? For many species, cellular survival does not strictly depend on peroxisomal functions; hence, their repertoire of proteins could change without detrimental consequences. Furthermore, peroxisomes represent a cellular one-way road. They import cargo, grow, divide, and are degraded but probably do not fuse regularly in their mature form (Islinger et al., 2012; Kim and Hettema, 2015; Germain and Kim, 2020). This may explain their tolerance to a plethora of proteins and the coexistence of multiple peroxisome variants in a single cell as evident for the Woronin body biogenesis in ascomycetes.

## Peroxisomes and mitochondria—striking similarities

### Dually targeted soluble proteins

Mitochondria and peroxisomes cooperate in versatile metabolic processes, exchange many molecules, and also share proteins required for fission and quality control (Schrader et al., 2015; Wanders et al., 2016). This peroxisome–mitochondria connection has been excellently discussed before (Fransen et al., 2017; Costello et al., 2018), and we only focus on several examples. Both organelles can be cellular sites for β-oxidation of fatty acids—while in several fungi and plants, β-oxidation occurs exclusively inside of peroxisomes, other fungi and animals harbor full sets of β-oxidation enzymes inside of both organelles (Maggio-Hall and Keller, 2004; Poirier et al., 2006; Goepfert and Poirier, 2007; Kretschmer et al., 2012b; Camões et al., 2015). An important difference between the compartments is the mechanisms they use for the import of proteins: While mitochondrial proteins often rely on N-terminal targeting signals and can be imported in an unfolded or flexible state (Backes and Herrmann, 2017), import into the peroxisomal matrix is different.

To exchange intermediates of the β-oxidation pathway, substitution of a coenzyme A moiety with carnitine is one possibility to shuttle acetyl and acyl groups as acetyl- or acylcarnitine units (Antonenkov and Hiltunen, 2012). Acetylcarnitine transferases are dually localized enzymes, and isoforms with different targeting signals have been reported, which can be generated *via* alternative transcriptional and translational start sites or differential splicing (Corti et al., 1994; Elgersma et al., 1995; Ueda et al., 1998; Houten et al., 2020). Within mitochondria, carnitine is replaced by coenzyme A to enable further metabolization (Houten et al., 2020).

A second cellular pathway enables the exchange of C2 units between peroxisomes and mitochondria, albeit more indirectly. Citrate can be generated from oxaloacetate *via* citrate synthases, which are not only part of the Krebs cycle but also part of the glyoxylate cycle, partially located inside of peroxisomes (Kim et al., 1986; Kunze et al., 2006). The citrate synthase Cit2 has overlapping functions with acetylcarnitine transferase Cat2p in *S. cerevisiae* and is contained in peroxisomes (Van Roermund et al., 1995; van Roermund et al., 1999; Swiegers et al., 2001; Shai et al., 2018). Of interest, Cit2p can be targeted to mitochondria, as well as to peroxisomes, and can compensate for the absence of mitochondrial citrate synthase Cit1p (Kim et al., 1986; Hoppins et al., 2011; Lee et al., 2011; Morgenstern et al., 2017). Citrate can be shuttled *via* dedicated transport proteins linking the mitochondrial matrix to the cytosol (Kaplan et al., 1995; Palmieri, 2004).

How are proteins distributed that do not come in different isoforms but contain a mitochondrial targeting signal at the N-terminus and a PTS1 at the C-terminus? Previously, it was discussed that these usually end up in mitochondria, because the N-terminal signal will exit the ribosome first, can directly interact with the mitochondrial import machinery, and thus will dominate a PTS1—a hierarchy of targeting signals is likely to exist (Kunze and Berger, 2015). Indeed, the mitochondrial ribosomal protein Mrp7p contains a functional PTS1 in *S. cerevisiae* but does not localize in peroxisomes presumably because the N-terminal signal is recognized first (Neuberger et al., 2004). However, several *bona fide* peroxisomal proteins such as the thiolase Tes1p or the catalase Cta1p have been identified in highly purified mitochondria of *S. cerevisiae* (Morgenstern et al., 2017).

**FIGURE 2:**
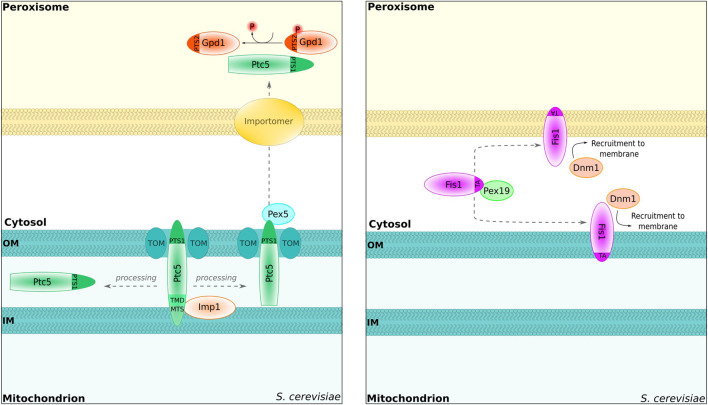
Proteins shared between peroxisomes and mitochondria. *S. cerevisiae* Ptc5p can reach the peroxisome *via* mitochondrial transit. The protein is proteolytically processed inside of the inner mitochondrial membrane by the peptidase Imp1 and subsequently translocated to peroxisomes in a Pex5-dependent manner [left; Stehlik et al. (2020)]. Inside of the peroxisome, Ptc5 dephosphorylates Gpd1 [left; Stehlik et al. (2020)]. TOM, translocator of the outer mitochondrial membrane. Importomer, complex for peroxisomal matrix protein import [Walter and Erdmann (2019)]. The tail-anchored protein Fis1 is involved in the fission process of peroxisomes and mitochondria [right; Kuravi et al. (2006)]. Targeting is regulated by Pex19 [Cichocki et al. (2018)]. Dnm1, dynamin-related GTPase for peroxisome fission Kuravi et al. (2006).

Another PTS1-containing protein—the protein phosphatase Ptc5p—has been recently shown to first target mitochondria where its N-terminus is proteolytically removed. Subsequently, the protein is translocated into the peroxisomal lumen *via* interaction with Pex5p (Stehlik et al., 2020) (Figure 2). Further proteins were also shown to be retargeted from mitochondria to the cytosol, e.g., *S. cerevisiae* fumarase (Stein et al., 1994; Yogev et al., 2010). The sorting of Ptc5p is different as the presence of Pex5p is critical for export from mitochondria, indicating an interaction between Ptc5p and Pex5p prior to full mitochondrial import. One target of Ptc5p in peroxisomes is Gpd1p (Stehlik et al., 2020), and dephosphorylation of Gpd1p increases its activity (Lee et al., 2012). In line with these data, a synthetic growth defect was observed for strains deleted for *PTC5* and *MDH3* (Costanzo et al., 2016; see the previous chapter on the redundancy of Gpd1p and Mdh3p). Together, these data suggest a network consisting of Ptc5p, Gpd1p, and Mdh3p to regulate peroxisomal NADH metabolism presumably in a manner adapting the different compartments to each other. In *A. thaliana*, the sorting of NAD(P)H dehydrogenases may resemble Ptc5p from *S. cerevisiae* as competing N- and C-terminal targeting signals for mitochondria and peroxisomes are involved (Carrie et al., 2008).

Targeting of proteins with competing targeting signals may represent a more generic approach to regulate the interaction and communication of compartments—the overexpression of several of these proteins increases the fraction of peroxisomes associated with mitochondria (Stehlik et al., 2020). In mammalian cells, the overexpression of the dual affinity protein ACBD2/ECI2 also enhances the proximity of peroxisomes and mitochondria (Fan et al., 2016). The protein Cnm1p with competing targeting signals at its termini can increase the attachment of mitochondria to the nuclear envelope in *S. cerevisiae*, suggesting a similar mechanism occurring between another pair of organelles (Eisenberg-Bord et al., 2021).

### Dual targeting of mitochondrial membrane proteins

In addition to luminal proteins, peroxisomes and mitochondria share a number of membrane proteins or membrane-associated proteins with various tasks (also see Fransen et al., 2017; Costello et al., 2018). A key process controlled by overlapping factors is fission. Both in *S. cerevisiae* and in mammalian cells, the C-terminally tail-anchored protein Fis1p/FIS1, dynamin-related GTPases, and additional shared proteins such as the WD40 repeat-containing protein Caf4p belong to the factors involved in the fission process (Koch et al., 2003; Praefcke and McMahon, 2004; Koch et al., 2005; Kuravi et al., 2006; Motley et al., 2008; Motley et al., 2015; Castro et al., 2018). C-terminally tail-anchored membrane proteins such as FIS1 tend to be dually localized, and it was shown that the hydrophobicity of the transmembrane domain together with changes in the charge of the luminal tail determines targeting efficiency and hence the subcellular distribution (Costello et al., 2017a). Remarkably, the peroxisomal targeting factor Pex19p is required for the correct sorting of Fis1p to mitochondria and to peroxisomes in *S. cerevisiae* (Cichocki et al., 2018) (Figure 2). How the distribution of proteins such as Fis1p is adapted to the needs of both organelles is not known yet.

Further proteins operating at mitochondria and peroxisomes are ATPases termed Msp1p in *S. cerevisiae* or ATAD in mammals, which can extract superfluous or mistargeted tail-anchored proteins (Chen Y.-C. et al., 2014; Okreglak and Walter, 2014; Weir et al., 2017). Of interest, a variety of PMPs can be removed from mitochondria *via* Msp1p/ATAD, suggesting a broader range of substrates (Nuebel et al., 2021). Furthermore, these authors observed that many peroxisomal proteins are not degraded or downregulated in the absence of preexisting peroxisomes, but functional PMP-containing protein complexes assemble on mitochondria, which causes unfavorable consequences such as the import of PTS1 cargo into mitochondria (Nuebel et al., 2021). Mitochondrial dysfunction in the absence of functional peroxisomes is emerging as one cause of symptoms in patients lacking peroxisomes (Wanders, 2014; Lismont et al., 2019; Schrader et al., 2020; Nuebel et al., 2021). The ubiquitin ligase MARCH5—another protein for quality control—was shown to localize to peroxisomes in addition to its known location at mitochondria in mammals. At peroxisomes, MARCH5 is involved in controlling selective autophagy (Zheng et al., 2021).

A multi-subunit protein structure associated with both organelles in fungi is the ERMES (ER–mitochondria encounter structure) complex, which has a role in tethering of mitochondria to the ER and to peroxisomes to form putative three-way junctions (Kornmann et al., 2009; Cohen et al., 2014; Ušaj et al., 2015; Kundu and Pasrija, 2020). Moreover, the ERMES regulating GTPase Gem1p can be found in peroxisomes and mitochondria of *S. cerevisiae* (Kornmann et al., 2011; Cichocki et al., 2018). In mammalian cells, the Gem1p ortholog MIRO1 is dually localized as well and has a role in recruiting the lipid transfer protein VPS13D probably involved in sustaining organellar growth and lipid exchange (Castro et al., 2018; Baldwin et al., 2021; Guillén-Samander et al., 2021). Thus, mitochondria and peroxisomes use an overlapping set of proteins to connect to the ER.

A different mode of transport to accomplish the dual localization of selected proteins is vesicular trafficking from one organelle to the other. Dynamin-independent carriers were proposed to transport the ubiquitin ligase MAPL from mitochondria to peroxisomes (Neuspiel et al., 2008). More recently, it was suggested that *de novo* peroxisome formation involves mitochondria-derived vesicles (Sugiura et al., 2017).

### Dual targeting and evolution of peroxisomes

All these data point to a close relationship of mitochondria and peroxisomes not only in terms of metabolism but also in terms of biogenesis, quality control, and turnover. This remarkable overlap might reflect peroxisome evolution as partially mitochondria-derived organelles (Bolte et al., 2015; Speijer, 2017). Are peroxisomes indeed outposts of mitochondria, and how can this be in line with the key role of the endoplasmic reticulum for peroxisome biogenesis (Joshi et al., 2017; see also in the next chapter)?

**FIGURE 3:**
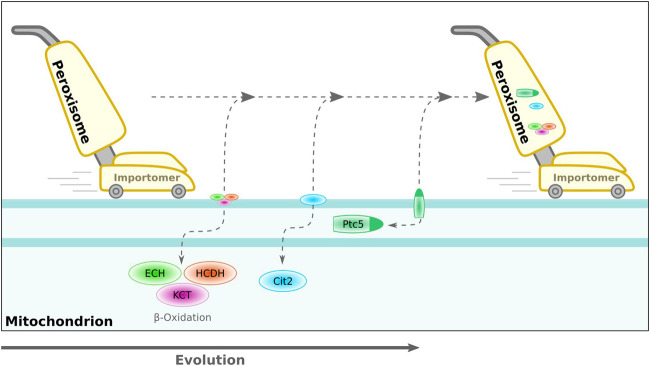
Hypothetical model for translocation of proteins from mitochondria to peroxisomes during evolution. Peroxisomes are indicated by a vacuum cleaner—this parallel is inferred from their unique import mode for folded proteins and the possible evolutionary origin of the importomer from an ER-derived quality control system (Gabaldón et al. (2006), Schlüter et al. (2006)). We speculate that over time, various mitochondrial enzymes developed into peroxisomal enzymes *via* a dually targeted intermediate. Sorting of the phosphatase Ptc5p from *S. cerevisiae* shows hallmarks for such an evolutionary scenario. ECH, enoyl-CoA hydratase; HCDH, 3-hydroxyacyl-CoA dehydrogenase; KCT, 3-ketoacyl-CoA-thiolase; Cit2, citrate synthase 2 (*S. cerevisiae*); Ptc5, PP2C-type phosphatase (*S. cerevisiae*).

We speculate that peroxisomes may have emerged from an ancient ER-derived quality control compartment involved in clearing specific mitochondrial proteins, especially under conditions of oxidative stress. In this perspective, the ERAD-related peroxisomal import machinery (Schliebs et al., 2010) could be regarded as an extraction machine for mitochondrial proteins working *in trans*. Particularly, the oxidative enzymes of the β-oxidation pathway may be a major burden for mitochondrial metabolism, which may explain why this pathway has been completely transferred to peroxisomes of several species, e.g., *S. cerevisiae* (Hiltunen et al., 2003; Poirier et al., 2006; Speijer, 2017). The indirect targeting of the phosphatase Ptc5p *via* mitochondrial transit (Stehlik et al., 2020) could be a snapshot or remnant of this evolutionary scenario. In this effect, peroxisomes may represent ancient molecular vacuum cleaners of the mitochondrial surface (Figure 3), which became autonomous over time by hitchhiking the mitochondrial division and quality control machinery. Further shared proteins might still be obscure—one study indicated a role for the dually localized mitofusin ortholog Fzo1 from *S. cerevisiae* for tethering peroxisomes to mitochondria (Shai et al., 2018).

## Proteins localized to peroxisomes and the endoplasmic reticulum

### The endoplasmic reticulum as a source for peroxisomal membrane

The endoplasmic reticulum (ER) is the major source of cellular lipids and has a key role in supporting the growth of the entire cell (Ferro-Novick et al., 2013). Early work already revealed intimate connections between this large-supply organelle and peroxisomes (Novikoff and Shin, 1964). Metabolites are exchanged between both compartments, e.g., during the biosynthesis of ether lipids (Wanders et al., 2016). In *S. cerevisiae*, the sorting of the multifunctional PMP Pex3—a key factor in regulating PMP import into peroxisomes—from the ER to peroxisomes was described (Hoepfner et al., 2005; Jansen and van der Klei, 2019). ER-derived vesicular carriers were reported to contain peroxisomal proteins, and machinery was uncovered that is relevant for the emergence of peroxisomal vesicles from the ER in yeasts (Titorenko et al., 2000; Lam et al., 2010; Agrawal et al., 2011; Van der Zand et al., 2012; Agrawal et al., 2016; Mast et al., 2018). Hence, vesicular trafficking from the ER represents one road for lipids and proteins to the peroxisome and also operates in mammalian cells (Kim et al., 2006; Aranovich et al., 2014; Sugiura et al., 2017). In *S. cerevisiae* and other yeasts, ER-resident microdomain-forming reticulon proteins play a critical role in tethering of preexisting peroxisomes (David et al., 2013; Mast et al., 2016; Wu et al., 2020; Ferreira and Carvalho, 2021). Interestingly, the same domains may be required for the budding of peroxisome precursors (Joshi et al., 2016; Joshi et al., 2018; Wang et al., 2018). As an alternative to vesicle formation, direct lipid transfer promotes the growth of peroxisomes and recruitment of lipid transfer proteins such as VPS13 is involved in peroxisome biogenesis both in yeasts and in mammals (Raychaudhuri and Prinz, 2008; Baldwin et al., 2021; Guillén-Samander et al., 2021; Yuan et al., 2022). How these mechanisms for lipid supply each contribute to peroxisome formation is an exciting question for future work. An intimate physical connection between peroxisomes and the ER is probably required for the correct activity of both mechanisms. This can, e.g., be supported through an interaction between the peroxisomal acyl-CoA binding protein ACBD5 and the ER membrane-associated protein VAPB in mammalian cells (Costello et al., 2017b; Hua et al., 2017).

### Proteins found in the endoplasmic reticulum and peroxisomes

Many PMPs are synthesized in the vicinity of the peroxisome of *S. cerevisiae* (Zipor et al., 2009; Dahan et al., 2022), and direct insertion into the peroxisomal membrane was demonstrated for several of them (Sacksteder et al., 2000; Fang et al., 2004; Jones et al., 2004; Matsuzaki and Fujiki, 2008; Yagita et al., 2013; Chen Y. et al., 2014). Interestingly, other PMPs are synthesized proximal to the ER and may reach the peroxisome *via* ER transit (Jan et al., 2014). For Pex3, dual localization to peroxisomes and the ER of *S. cerevisiae* was shown by fractionation experiments (Mast et al., 2016). Hence, the ER is not only an intermediate but also a relevant steady-state location for certain PMPs, indicating functions beyond ER transit. Indeed, in mammalian cells ER-localized PEX3 is involved in the sorting of a protein designated for lipid droplets (Schrul and Kopito, 2016). In addition to PEX3, the soluble targeting factor PEX19 is associated with the ER and involved in targeting reticulon-homology proteins to this organelle besides its prominent function in peroxisome biogenesis (Yamamoto and Sakisaka, 2018; Zimmermann et al., 2021).

The coat protein I (COPI) complex localizes primarily along the organelles of the secretory pathway where it is involved in retrograde transport (Barlowe and Miller, 2013). In addition, this protein assembly can be associated with peroxisomes and also with mitochondria, but the molecular function of the localization at these organelles is not fully understood (Passreiter et al., 1998; Barlowe and Miller, 2013; David et al., 2013; Zabezhinsky et al., 2016). Remarkably, COPI-dependent sorting of a viral protein from peroxisomes to the ER was reported (McCartney et al., 2005). The small GTPase Arf1p—a major regulator of COPI—shows multiple subcellular localizations including peroxisomes and mitochondria in *S. cerevisiae* (Ackema et al., 2014; Yofe et al., 2017). So far, the role of COPI-dependent vesicular trafficking for peroxisome biogenesis is elusive although in *S. cerevisiae,* COPI components can be purified together with the subdomain-forming protein Pex30p, which is involved in peroxisome formation (David et al., 2013). Pex30p is also found at sites of lipid droplet formation in *S. cerevisiae*, and COPI was shown to be involved in formation of this organelle in *Drosophila* cells (Wilfling et al., 2014; Joshi et al., 2018; Wang et al., 2018; Ferreira and Carvalho, 2021). Further research is required to understand how key factors of the secretory pathway are dynamically distributed between the different organelles and how this coordinates organelle biogenesis at different sites. Nevertheless, the discussed examples emphasize that the ER and peroxisomes are possible alternative destinations of several factors required for the proper maintenance of each.

Another interesting example of dual targeting was described in *A. thaliana—*a purple acid phosphatase-containing competing targeting signals is localized to the ER and to peroxisomes (Kataya et al., 2016). Various other phosphatases with noncanonical PTS1 motifs were identified in this study that may reside inside of peroxisomes and in additional compartments including the nucleus (Kataya et al., 2016).

### Dual targeting to the nucleus and peroxisomes

Different types of pathogenic attack trigger the nuclear localization of plant catalases (Inaba et al., 2011; Zhang M. et al., 2015). A recent study in *A. thaliana* uncovered that catalase is localized both in peroxisomes and in the nucleus even in the absence of any infection (Al-Hajaya et al., 2022). Regulation of dual targeting *via* metabolites such as H_2_O_2_ is likely to operate in plants similar to what was observed in mammals (Okumoto et al., 2020). Peroxisomes together with mitochondria have a key role in controlling ROS production, which is important for cellular signaling and needs to be maintained on a level acceptable for the integrity of cells, especially of the nucleus (Fransen et al., 2017; Sies, 2017; Li et al., 2021; Paradis et al., 2021).

Interestingly, the hypoxia-inducible transcription factor Hif1 and hydroxylases regulating its activity are found in peroxisomes and mitochondria (Khan et al., 2006; Li et al., 2019). Sequestration upon reoxygenation was suggested as a possible biological function of peroxisomal targeting (Khan et al., 2006).

### Proteins shared by lipid droplets and peroxisomes

Lipid droplets are sites of cellular fat storage and mobilization. Their biosynthesis and function are both intricately linked to peroxisomes. Both organelles require overlapping proteins for biogenesis, e.g., the lipodystrophy protein seipin and other factors including Pex30p (Szymanski et al., 2007; Salo et al., 2016; Joshi et al., 2017; Joshi et al., 2018; Wang et al., 2018). The targeting factor PEX19 and ER-localized PEX3 facilitate the insertion of the membrane protein UBXD8 into a subdomain of the ER membrane, which turned out as a prerequisite for subsequent transfer to lipid droplets in mammalian cells (Schrul and Kopito, 2016). Upon depletion of PEX19, UBXD8 appeared predominantly in mitochondria. Of interest, a proper targeting of UBXD8 requires C-terminal farnesylation of PEX19 (Schrul and Kopito, 2016).

**FIGURE 4:**
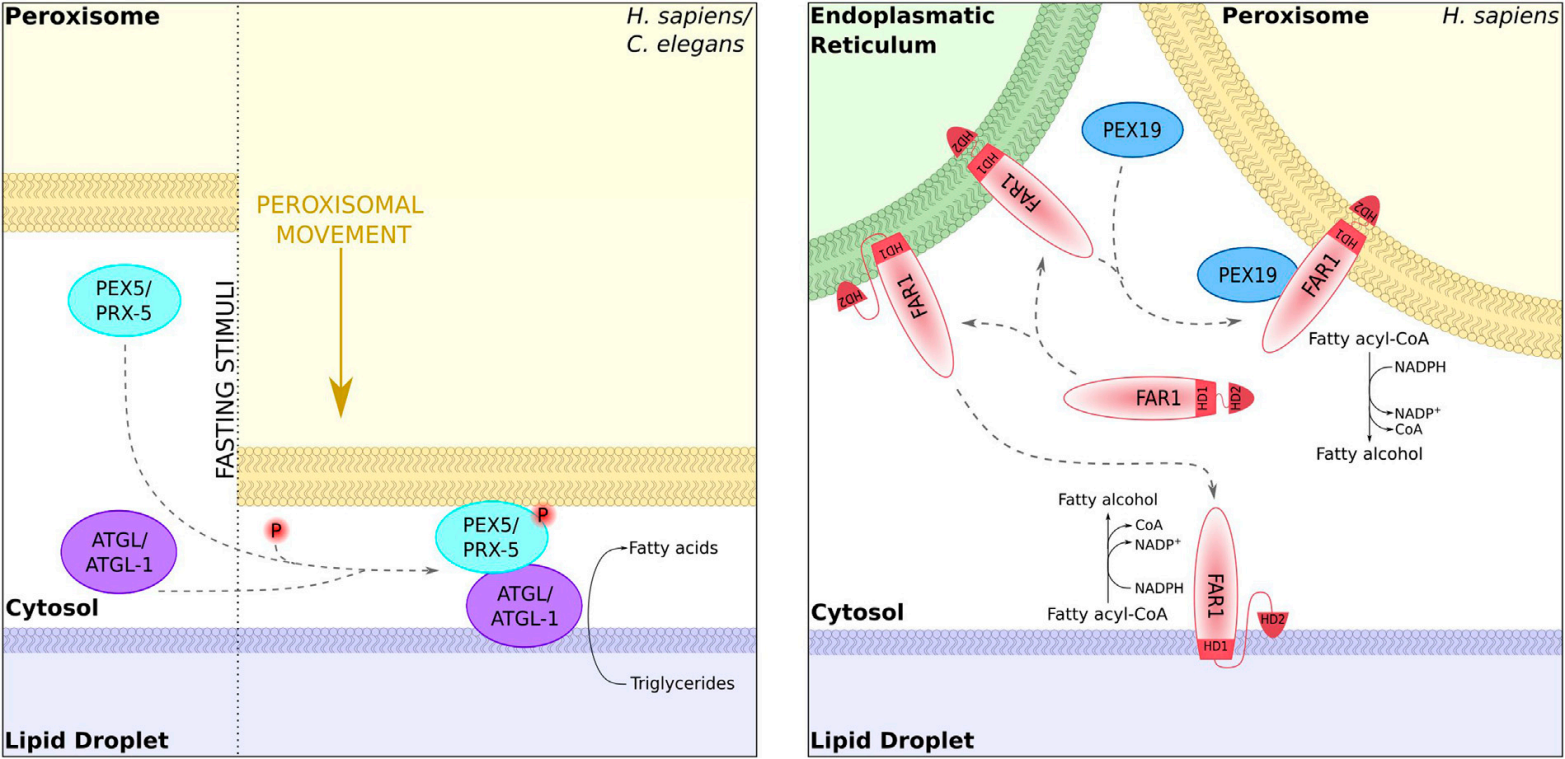
Dual targeting to peroxisomes and lipid droplets. The lipases ATGL-1 (*Caenorhabditis elegans*) and ATGL (mammalian cells) can target lipid droplets in a Pex5-dependent manner at sites of organelle contact [left; Kong et al. (2020)]. The fatty acid reductase Far1 is dually localized to peroxisomes and lipid droplets. Localization to one or the other organelle requires different topologies of the hydrophobic domains [right; Exner et al. (2019)].

Peroxisomes and lipid droplets are physically connected, and membrane protrusions emerging from peroxisomes have been shown to attach to lipid droplets in *S. cerevisiae* (Binns et al., 2006). In *A. thaliana*, a dually localized lipase can be sorted from peroxisomes to lipid droplets *via* similar protrusions in the course of fat mobilization during seed germination (Thazar-Poulot et al., 2015). In a similar way, in fasting animals triglyceride lipases are translocated from peroxisomes to lipid droplets depending on the targeting factor PEX5. This process was proposed to involve sites of organelle contact (Kong et al., 2020) (Figure 4).

Since peroxisomes are enclosed by a lipid bilayer and lipid droplets only by a monolayer, the dual localization of membrane proteins is likely to depend on dual topology. This was demonstrated for mammalian fatty acyl-CoA reductase 1, which is targeted to peroxisomes and lipid droplets and transits *via* the ER (Exner et al., 2019) (Figure 4).

### What about the remaining cell?

Knowledge about proteins destined for peroxisomes and the plasma membrane or peroxisomes and endosomes is limited. It has been described that fungal peroxisomes hitchhike on motile endosomes to move through fungal hyphae (Guimaraes et al., 2015; Salogiannis et al., 2016). In *A. nidulans*, the association between endosomes and peroxisomes is mediated by the linker protein PxdA (Salogiannis et al., 2016). Proteins required for the maturation of endosomes such as members of the ESCRT (endosomal sorting complex required for transport) pathway (Henne et al., 2011) are involved in peroxisome biogenesis (Mast et al., 2018). Of interest, recent work revealed intraluminal vesicles inside of plant peroxisomes, which might also require the function of the ESCRT pathway, hinting at some parallels to multi-vesicular bodies (Wright and Bartel, 2020).

### How to choose between the different destinations?

Insights into the molecular mechanisms that regulate the distribution of proteins among their different destinations are of interest to understand communication and homeostasis of cellular compartments. Studies on the dual localization of catalase in mammalian cells could represent a blueprint—it was shown that an elevated cytosolic concentration of H_2_O_2_ provokes cytosolic retention of this detoxifying enzyme (Walton et al., 2017). Recent studies showed phosphorylation of the peroxisomal membrane Pex14 in response to oxidative stress and during mitosis, which reduces the peroxisomal import of catalase to a greater extent than the import of other tested matrix proteins (Okumoto et al., 2020; Yamashita et al., 2020). Modification of the peroxisomal import machinery, hence, has an important role to adapt the peroxisomal and cytosolic protein composition to cellular demands. In fungi, the peroxisomal import of catalase is exceptional, and it relies on a noncanonical PTS1 and a second unusual peroxisomal targeting signal closer to the N-terminus (Kragler et al., 1993; Williams et al., 2012). Substitution of the C-terminus with a canonical targeting signal lowers catalase activity and leads to the aggregation of the enzyme (Williams et al., 2012). In *S. cerevisiae*, peroxisomal localization of the glyoxylate cycle enzyme citrate synthase 2 is reduced upon expression of a version of Pex14 mimicking a phosphorylated state (Schummer et al., 2020). Phosphorylation of the targeting factor Pex5 has been implicated in the regulation of pexophagy upon stress exposure in mammalian cells and occurs in different species (Zhang J. et al., 2015; Oeljeklaus et al., 2016). How phosphorylation of Pex5 modulates the import of specific cargo besides regulating peroxisome breakdown remains to be established. Redox regulation at an N-terminal cysteine residue is another way to control Pex5 activity (Ma et al., 2013).

Phosphorylation of dually localized cargo proteins is also involved in regulating their intracellular distribution (Figure 1). *S. cerevisiae* glycerol-3-phosphate dehydrogenase Gpd1 is phosphorylated in the vicinity of the PTS2 (Jung et al., 2010). While phosphomimetic variants of Gpd1 are efficiently targeted to the peroxisome, variants resembling non-phosphorylated Gpd1 are retained in the cytosol.

Many fungal species encode at least two versions of the PTS1 receptor Pex5 (Kiel et al., 2006). The expression of the Pex5 paralog Pex9 is induced upon incubation of *S. cerevisiae* cells in oleic acid medium and regulates the import of a subset of peroxisomal matrix proteins with specific targeting signals (Effelsberg et al., 2016; Yifrach et al., 2016; Yifrach et al., 2022). In the corn smut fungus *Ustilago maydis*, two Pex5 paralogs with different cargo selectivity are required for peroxisome function (Ast et al., 2022). The concentration or availability of different targeting factors at peroxisomes and at other organelles may emerge as an additional regulatory device to control the subcellular distribution of proteins.

## Conclusion

Knowledge about proteins with various cellular destinations significantly increased in the last years. It probably will continue to grow rapidly as sophisticated approaches become more and more available, which allow the tracking of minor or transient destinations. Careful investigation to confirm whether dual or multiple localizations are of biological significance will be required. Targeted disruption of sorting signals leaving the overall protein function intact is one approach to reveal a function inside of a particular organelle (Kunze et al., 2002; Freitag et al., 2012).

The mechanisms to achieve dual or multiple targeting are highly variable, and how they are embedded in the regulatory circuits of the cell is not established in many of the cases. Proteins with different destinations may further emerge as critical factors regulating organellar interplay in terms of metabolism and molecule exchange, as well as in terms of biogenesis and proliferation. Thus, a better characterization of how the shared proteins are distributed and how this is regulated will improve our understanding of eukaryotic cells.
